# Supplementary material for: A heterocyte glycolipid-based calibration to reconstruct past continental climate change
Source: Nat Commun. 2021 Apr 23;12:2406. doi: 10.1038/s41467-021-22739-3 (PMC8065054; doi:10.1038/s41467-021-22739-3)
Supplement: Supplementary file 1 — Supplementary Information [file 41467_2021_22739_MOESM1_ESM.pdf]

Supplementary Information

Supplementary Fig. 1 Chemical structures of heterocyte glycolipids detected in East African lakes as well as polar to tropical lakes and ponds. (**Ia**) 1-(O-hexose)-3,25-hexacosanediol (HG_{26} diol); (**Ib**) 1-(O-hexose)-3-keto-25-hexacosanol (HG_{26} keto-ol); (**Ic**) 1-(O-hexose)-3,27-octacosanediol (HG_{28} diol); (**Id**) 1-(O-hexose)-3-keto-27-octoacosanol (HG_{28} keto-ol); (**Ie**) 1-(O-hexose)-3,29-triacontanediol (HG_{30} diol); (**If**) 1-(O-hexose)-3-keto-29-triacontanol (HG_{30} keto-ol); (**Ig**) 1-(O-hexose)-3,25,27-octacosanetriol (HG_{28} triol); (**Ih**) 1-(O-hexose)-3-keto-25,27-octacosanediol (HG_{28} keto-diol); (**Ii**) 1-(O-hexose)-3,27,29-triacontanetriol (HG_{30} triol); (**Ij**) 1-(O-hexose)-3-keto-27,29-triacontanediol (HG_{30} keto-diol); (**Ik**) 1-(O-hexose)-3,29,31-dotriacontanetriol (HG_{32} triol); (**Ij**) 1-(O-hexose)-3-keto-29,31-dotriacontanediol (HG_{32} keto-diol); (**IIa**) deoxyhexose-3,25-hexacosanediol (DE HG_{26} diol); (**IIIa**) pentose-3,25-hexacosanediol (P HG_{26} diol).

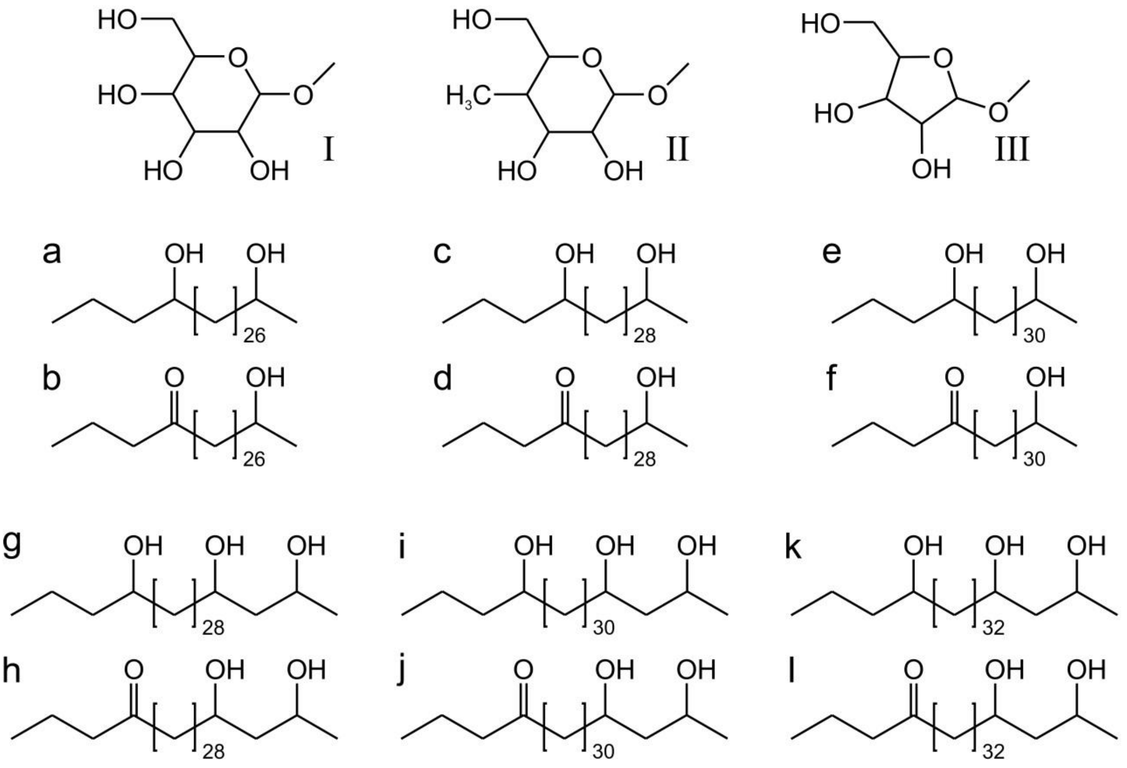

Supplementary Table 1 . Geographical, environmental and physiochemical parameters of East African lakes as well as other lakes and ponds from polar to tropical latitudes.

Lake	Abbreviation	Country	Latitude	Longitude	Elevation	Maximum Depth	Surface Area	MAAT	SWT	BWT	SW pH	BW pH	SW DO	BW DO	Conductivity	TP	TN	DOC	Chl a	TOC	TON
					(masl)	(m)	(m^2)	(°C)	(°C)	(°C)			(mg/l)	(mg/l)	(µS/cm)	(µg/l)	(µg/l)	(mg/l)	(µg/l)	(wt%)	
East African lakes																					
Albert	Al	Uganda	1.683	30.917	615	58	5.7E+09	26.8	27.9	27.9	9.0	9.0	--	3.0	770	165.0	23.0	--	--	5.1	0.005
Bandasa	Ba	Kenya	-0.157	37.449	2938	0	5.4E+03	12.8	17.2	17.2	8.1	8.1	2.5	2.5	21	481.5	3159.0	14.2	51.3	5.1	0.004
Bigata	Bi	Uganda	0.307	29.883	3983	18	1.4E+04	4.2	9.0	5.4	5.7	5.9	7.3	0.2	8	15.7	407.8	11.8	2.4	34.0	0.027
Bugwagi	Bu	Uganda	-0.200	30.183	1061	85	6.0E+05	24.0	26.5	24.7	8.8	7.2	6.0	0.3	439	18.0	334.8	4.5	--	11.9	0.009
Bujuku	Bk	Uganda	0.378	29.893	3891	14	7.9E+04	4.6	8.4	6.9	6.7	6.0	6.7	2.3	50	10.7	270.4	4.7	2.1	43.2	0.024
Bukurungu East	Be	Uganda	0.400	29.900	3801	17	3.0E+04	5.4	9.7	6.7	3.8	4.3	7.0	0.2	13	24.5	362.0	7.7	7.9	36.2	0.027
Edward	Ed	Uganda	-0.246	29.750	910	29	2.3E+09	25.1	26.8	25.8	8.9	8.1	6.5	2.0	840	127.0	23.5	--	7.2	14.1	--
Eldoret Nakuru 1a	El 1	Kenya	0.440	35.305	2185	1	1.0E+03	15.8	14.1	14.1	6.9	6.9	4.0	3.0	196	--	--	--	26.5	8.4	--
Eldoret Nakuru 2	El 2	Kenya	0.361	35.351	2217	2	5.0E+02	15.6	14.5	14.5	6.9	6.9	3.4	3.4	171	--	--	--	--	0.4	--
Eldoret Nakuru 3	El 3	Kenya	0.333	35.365	2214	1	3.0E+02	15.7	16.3	16.3	7.0	7.0	4.0	3.0	221	--	--	--	--	1.9	--
Ellis	Es	Kenya	-0.124	37.401	3474	16	1.1E+05	10.1	12.6	11.5	7.4	7.3	6.1	4.2	41	8.0	412.0	5.4	4.6	25.4	0.024
Enchanted	En	Kenya	-0.172	37.336	4237	33	4.9E+04	6.3	8.2	7.9	7.6	6.6	6.2	5.9	32	5.5	66.0	7.2	1.6	7.5	0.009
Hausburg Tarn	Hat	Kenya	-0.145	37.301	4371	11	1.7E+04	5.6	7.9	6.5	8.4	7.0	6.7	7.0	24	5.8	131.0	2.3	5.4	8.0	0.010
Hohnell	Ho	Kenya	-0.183	37.290	4212	9	1.1E+05	6.4	7.9	7.4	7.3	6.4	6.5	6.0	31	50.0	208.0	3.4	13.8	6.7	0.008
Hut Tarn	Hut	Kenya	-0.157	37.299	4504	8	2.0E+04	4.9	5.7	5.0	7.6	6.5	6.9	6.9	20	8.2	125.0	3.6	3.2	13.6	0.016
Ibanda Pool	Ib	Uganda	0.360	30.018	1735	5	3.3E+03	18.4	15.3	14.8	7.9	8.0	8.4	8.4	64	50.0	594.0	--	--	3.9	0.003
Karolero	Ka	Uganda	-0.333	30.033	1114	15	5.1E+04	23.5	26.7	25.5	8.2	7.3	5.1	2.2	168	45.2	374.0	4.2	--	13.9	0.019
Kasenda	Ks	Uganda	0.433	30.283	1260	14	9.3E+04	22.3	25.8	23.6	8.5	6.9	7.7	0.0	317	40.9	662.5	4.0	--	--	--
Kasirya	Kr	Uganda	-0.250	30.133	1235	43	8.7E+04	22.3	26.3	23.2	8.7	6.6	4.9	0.0	302	18.3	152.7	--	--	17.4	0.014
Katalin	Kt	Kenya	0.633	35.483	2337	5	1.5E+05	14.9	19.5	19.5	6.6	6.6	7.6	0.3	78	--	--	--	--	4.7	--
Kibengo	Kb	Uganda	-0.067	30.167	914	6	8.8E+05	25.1	27.7	26.5	9.8	9.3	7.0	2.2	220	93.8	2108.0	12.6	--	11.4	--
Kifuruka	Ki	Uganda	0.483	30.283	1402	4	1.3E+05	21.1	23.4	21.9	7.8	7.4	5.1	1.1	359	78.0	497.2	4.1	3.3	40.3	0.040
Kisibendi	Kn	Uganda	0.383	30.300	1126	5	--	23.3	25.0	22.7	9.3	6.2	5.7	0.0	277	300.1	1952.5	--	--	30.6	0.051
Kitere	Ke	Uganda	0.400	30.250	1252	51	9.8E+04	23.1	26.7	23.8	9.0	7.1	4.0	0.0	711	130.1	574.3	--	--	8.3	0.006
Kyanga	Ky	Uganda	0.400	30.233	1122	57	1.1E+05	23.4	26.9	24.1	9.4	7.3	8.2	0.1	1055	114.0	41.0	7.6	--	10.6	--
Kyaninga	Kg	Uganda	0.700	30.300	1531	58	2.4E+05	20.1	23.8	21.8	8.3	6.7	6.4	0.2	407	11.8	103.6	1.9	--	14.2	--
Large Hall Tarn	Lht	Kenya	-0.143	37.346	4297	8	9.3E+03	6.0	8.7	7.9	6.9	7.2	6.2	6.7	10	17.5	262.0	4.8	3.4	14.1	0.012
Lower Kachope	Lka	Uganda	0.334	29.872	3841	13	8.1E+03	5.1	8.4	7.4	6.7	6.0	7.1	5.2	23	14.0	475.4	9.1	3.5	26.4	--
Lower Kitandara	Lki	Uganda	0.349	29.887	3989	12	2.9E+04	4.3	6.5	5.9	7.3	6.9	8.3	7.5	30	9.1	215.0	3.3	4.0	21.2	--
Mahuhura	Ma	Uganda	0.433	30.267	1254	70	1.9E+05	20.5	24.6	23.3	9.0	8.0	3.6	0.0	603	232.9	543.9	--	--	28.7	0.026
Mbajo	Mb	Uganda	0.450	30.267	1234	35	8.0E+04	22.5	24.5	23.1	8.6	6.9	3.6	0.0	504	21.1	523.9	--	--	21.2	0.015
Middle Kachope	Mka	Uganda	0.333	29.867	3843	3	1.0E+04	5.1	8.7	7.3	6.9	6.6	7.6	6.6	25	13.6	457.8	10.1	4.5	21.0	0.021
Murabio	Mu	Uganda	-0.350	30.033	1100	15	1.6E+05	23.6	26.7	22.6	8.0	7.2	4.0	0.0	718	21.7	322.2	2.8	--	30.6	0.030
Murusi	Mr	Uganda	0.433	30.283	1217	60	2.1E+05	22.5	26.1	22.5	8.5	6.6	7.0	0.1	374	11.1	309.5	3.5	--	15.6	0.010
Mutinda	Mt	Uganda	0.274	29.928	3507	2	3.0E+01	6.2	7.7	7.7	6.3	6.3	--	--	18	59.8	515.2	6.7	0.2	8.3	--
Njarayabana	Nj	Uganda	0.433	30.250	1184	37	1.2E+05	22.9	25.5	23.4	8.8	7.8	6.1	0.1	861	795.6	498.6	3.0	--	6.3	0.005
Nyamswiga	Ny	Uganda	0.517	30.283	1463	40	2.9E+04	20.7	23.5	20.5	7.8	7.8	3.8	0.0	320	206.0	234.9	--	16.8	17.2	0.015
Nyamugosani	Na	Uganda	0.417	30.233	1234	37	1.1E+05	22.5	26.8	24.5	8.8	8.5	6.3	0.2	981	337.0	285.7	3.5	--	7.0	0.005
Nyantonde	Nt	Uganda	0.483	30.283	1387	180	1.3E+05	21.2	23.7	21.3	8.8	6.9	6.9	0.0	438	12.1	259.1	--	4.6	15.2	0.016
Nyungu	Nu	Uganda	-0.250	30.100	1220	26	1.4E+05	22.6	24.7	22.3	9.3	7.6	8.9	0.0	452	263.6	1498.7	--	--	8.4	--
Oblong Tarn	Ot	Kenya	-0.144	37.301	4376	3	1.1E+04	5.6	8.4	8.0	7.1	6.4	6.1	6.6	25	6.1	155.0	5.8	2.5	10.8	0.014
Saka	Sk	Uganda	0.700	30.233	1543	8	2.7E+05	20.0	22.7	20.7	7.6	6.3	9.1	0.0	612	80.8	895.0	--	--	38.0	--
Speke	Sp	Democractic Republic of the Congo	0.405	29.881	4235	17	3.9E+04	3.0	5.8	5.3	5.9	5.2	8.0	5.0	12	12.1	265.5	5.0	3.4	16.3	--
Tanganyika	Ta	Tanzania	-4.872	29.617	773	110	3.3E+10	23.8	25.7	24.5	9.1	8.8	7.2	2.3	680	6.2	15.0	--	1.2	5.8	--
Upper Kachope	Uka	Uganda	0.332	29.893	3961	11	4.6E+04	4.9	7.8	6.5	6.9	6.8	7.8	6.3	19	14.9	379.3	7.0	9.9	18.5	--
Upper Kitandara	Uki	Uganda	0.353	29.887	4018	14	3.3E+04	4.9	5.8	4.9	7.5	6.9	8.6	7.6	25	6.6	243.8	2.8	4.7	32.0	0.022
Other lakes and ponds																					
Conophyton Pond	CP	Antarctica	-77.846	-166.668	--	--	--	--	1.0	--	9.2	--	--	--	1480	--	--	--	--	--	--
Orange Pond	OP	Antarctica	-77.846	-166.668	--	--	--	--	2.5	--	9.7	--	--	--	1541	--	--	--	--	--	--
Laguna Potrok Aike	Pa	Argentina	-51.963	-70.379	113	100	7.6E+06	--	9.5	--	--	--	--	--	--	--	--	--	--	--	--
Lake Constance	Co	Germany	47.583	9.467	395	251	5.4E+08	--	20.6	--	--	--	--	--	--	--	--	--	--	--	--
Lake Schreventeich[1]	St	Germany	54.327	10.121	23	3	3.8E+05	--	15.7	--	7.5	--	4.9	--	--	--	--	--	--	--	--
Lake Lading	La	Indonesia	-8.009	113.313	324	9	3.1E+04	25.6	28.7	27.1	7.4	7.4	6.9	0.0	586	--	--	--	--	--	--
Lake Klakah	Kl	Indonesia	-7.986	113.272	230	26	4.1E+05	26.1	29.5	26.6	7.5	6.7	7.5	0.0	326	--	--	--	--	--	--
Lake Towuti	To	Indonesia	-2.750	121.121	293	203	5.6E+09	--	29.8	28.4	7.8	7.6	5.1	0.0	210	--	--	--	0.5	4.5	0.210

[1]Bauersachs et al. (2015)

Supplementary Table 2. Fractional abundances of the total suite of heterocyte glycolipids in surface sediments of East African lakes as well as lakes and ponds of polar to tropical latitudes.

Lake	Abbreviation	Country	DE HG_{26} diol	P HG_{26} diol	HG_{26} keto-ols		HG_{26} diols				HG_{28} keto-ols		HG_{28} diols		HG_{28} keto-diols			HG_{28} triols				HG_{30} keto-ols		HG_{30} diols		HG_{30} keto-diols			HG_{30} triols			HG_{32} keto-diols		HG_{32} triols		
			I	I	I	II	I	II	III	IV	I	II	I	II	I	II	III	I	II	III	IV	I	II	I	II	I	II	III	I	II	III	I	II	I	II	III
East African lakes																																				
Albert	Al	Uganda	0.00	0.00	0.01	0.00	0.83	0.02	0.00	0.00	0.00	0.00	0.08	0.00	0.00	0.00	0.00	0.00	0.00	0.00	0.00	0.00	0.00	0.00	0.00	0.00	0.00	0.00	0.04	0.00	0.00	0.00	0.00	0.00	0.00	0.00
Bandasa	Ba	Kenya	0.00	0.00	0.02	0.09	0.37	0.08	0.00	0.00	0.00	0.04	0.04	0.09	0.00	0.05	0.00	0.17	0.00	0.00	0.00	0.00	0.00	0.00	0.00	0.00	0.02	0.00	0.02	0.01	0.00	0.00	0.00	0.00	0.00	0.00
Bigata	Bi	Uganda	0.00	0.00	0.02	0.11	0.24	0.06	0.00	0.00	0.01	0.03	0.03	0.09	0.00	0.11	0.00	0.03	0.11	0.01	0.00	0.00	0.00	0.00	0.00	0.00	0.08	0.00	0.05	0.01	0.00	0.00	0.00	0.00	0.00	0.00
Bugwagi	Bu	Uganda	0.00	0.00	0.03	0.01	0.72	0.10	0.00	0.00	0.01	0.00	0.07	0.01	0.00	0.00	0.00	0.00	0.01	0.00	0.00	0.00	0.00	0.00	0.00	0.00	0.00	0.00	0.01	0.00	0.00	0.00	0.00	0.00	0.01	0.00
Bujuku	Bk	Uganda	0.00	0.02	0.02	0.13	0.29	0.09	0.00	0.00	0.01	0.03	0.03	0.04	0.00	0.09	0.02	0.00	0.05	0.07	0.00	0.00	0.00	0.00	0.00	0.00	0.04	0.01	0.05	0.02	0.00	0.00	0.00	0.00	0.00	0.00
Bukurungu East	Be	Uganda	0.00	0.01	0.03	0.15	0.35	0.12	0.00	0.00	0.01	0.02	0.07	0.03	0.00	0.06	0.01	0.01	0.04	0.06	0.00	0.00	0.00	0.00	0.00	0.00	0.01	0.00	0.02	0.01	0.00	0.00	0.00	0.00	0.00	0.00
Edward	Ed	Uganda	0.00	0.00	0.02	0.00	0.49	0.07	0.00	0.00	0.01	0.00	0.29	0.03	0.00	0.00	0.00	0.00	0.02	0.00	0.00	0.00	0.00	0.01	0.00	0.00	0.00	0.00	0.05	0.00	0.00	0.00	0.00	0.00	0.00	0.00
Eldoret Nakuru 1a	El 1	Kenya	0.00	0.01	0.03	0.05	0.22	0.03	0.00	0.00	0.01	0.03	0.07	0.04	0.00	0.00	0.00	0.00	0.01	0.01	0.00	0.00	0.00	0.00	0.00	0.03	0.09	0.00	0.29	0.07	0.01	0.00	0.00	0.00	0.00	0.00
Eldoret Nakuru 2	El 2	Kenya	0.00	0.00	0.01	0.06	0.38	0.13	0.00	0.00	0.00	0.00	0.00	0.00	0.00	0.01	0.00	0.00	0.02	0.01	0.00	0.00	0.00	0.00	0.00	0.00	0.12	0.00	0.18	0.06	0.01	0.00	0.00	0.00	0.00	0.00
Eldoret Nakuru 3	El 3	Kenya	0.00	0.00	0.01	0.04	0.25	0.01	0.00	0.00	0.00	0.01	0.03	0.02	0.00	0.03	0.00	0.31	0.01	0.00	0.00	0.00	0.00	0.00	0.00	0.01	0.05	0.00	0.13	0.06	0.01	0.00	0.00	0.00	0.00	0.00
Ellis	Es	Kenya	0.00	0.00	0.01	0.08	0.32	0.03	0.00	0.00	0.00	0.06	0.15	0.02	0.00	0.01	0.00	0.00	0.02	0.01	0.01	0.00	0.00	0.00	0.00	0.01	0.12	0.00	0.11	0.05	0.00	0.00	0.00	0.00	0.00	0.00
Enchanted	En	Kenya	0.00	0.00	0.01	0.19	0.35	0.06	0.00	0.00	0.00	0.01	0.03	0.03	0.00	0.04	0.00	0.00	0.03	0.02	0.00	0.00	0.00	0.00	0.00	0.00	0.10	0.01	0.09	0.04	0.00	0.00	0.00	0.00	0.00	0.00
Hausburg Tarn	Hat	Kenya	0.00	0.00	0.00	0.01	0.05	0.02	0.00	0.00	0.00	0.00	0.00	0.03	0.00	0.09	0.00	0.04	0.01	0.00	0.00	0.00	0.00	0.00	0.00	0.00	0.56	0.00	0.16	0.03	0.00	0.00	0.00	0.00	0.00	0.00
Hohnell	Ho	Kenya	0.00	0.00	0.02	0.28	0.40	0.02	0.00	0.00	0.00	0.01	0.01	0.01	0.00	0.05	0.00	0.06	0.01	0.00	0.00	0.00	0.00	0.00	0.00	0.01	0.02	0.00	0.01	0.11	0.00	0.00	0.00	0.00	0.00	0.00
Hut Tarn	Hut	Kenya	0.00	0.00	0.01	0.09	0.31	0.06	0.00	0.00	0.00	0.02	0.04	0.02	0.00	0.03	0.00	0.04	0.02	0.00	0.00	0.00	0.00	0.00	0.00	0.00	0.15	0.00	0.16	0.05	0.00	0.00	0.00	0.00	0.00	0.00
Ibanda Pool	Ib	Uganda	0.00	0.01	0.01	0.07	0.27	0.02	0.00	0.00	0.01	0.03	0.08	0.03	0.03	0.07	0.00	0.00	0.15	0.07	0.00	0.00	0.00	0.00	0.00	0.00	0.03	0.01	0.08	0.03	0.00	0.00	0.00	0.00	0.00	0.00
Karolero	Ka	Uganda	0.00	0.00	0.00	0.00	0.07	0.01	0.00	0.00	0.01	0.04	0.68	0.06	0.00	0.00	0.00	0.00	0.00	0.00	0.00	0.00	0.00	0.01	0.00	0.00	0.00	0.00	0.09	0.00	0.00	0.00	0.00	0.01	0.00	0.00
Kasenda	Ks	Uganda	0.00	0.00	0.01	0.00	0.33	0.04	0.00	0.00	0.02	0.00	0.45	0.05	0.00	0.00	0.00	0.00	0.01	0.00	0.00	0.00	0.00	0.01	0.00	0.00	0.00	0.00	0.02	0.01	0.00	0.00	0.00	0.01	0.00	0.00
Kasirya	Kr	Uganda	0.00	0.00	0.02	0.00	0.42	0.05	0.00	0.00	0.02	0.00	0.37	0.05	0.00	0.00	0.00	0.01	0.00	0.00	0.00	0.00	0.00	0.01	0.00	0.00	0.00	0.00	0.01	0.00	0.00	0.00	0.00	0.01	0.00	0.00
Katalin	Kt	Kenya	0.00	0.01	0.01	0.04	0.05	0.18	0.00	0.00	0.01	0.02	0.03	0.01	0.00	0.00	0.00	0.48	0.01	0.01	0.00	0.00	0.04	0.00	0.03	0.00	0.02	0.00	0.04	0.01	0.00	0.01	0.00	0.01	0.00	0.00
Kibengo	Kb	Uganda	0.00	0.00	0.00	0.00	0.39	0.04	0.00	0.00	0.01	0.00	0.40	0.04	0.00	0.00	0.00	0.00	0.01	0.00	0.00	0.00	0.00	0.02	0.00	0.00	0.00	0.00	0.08	0.00	0.00	0.00	0.00	0.00	0.00	0.00
Kifuruka	Ki	Uganda	0.00	0.00	0.02	0.00	0.28	0.05	0.00	0.00	0.01	0.04	0.34	0.08	0.00	0.00	0.00	0.00	0.01	0.00	0.00	0.00	0.00	0.01	0.00	0.00	0.01	0.00	0.07	0.02	0.00	0.00	0.03	0.01	0.01	0.00
Kisibendi	Kn	Uganda	0.00	0.00	0.00	0.00	0.01	0.00	0.00	0.00	0.01	0.03	0.49	0.02	0.00	0.00	0.00	0.00	0.00	0.00	0.00	0.00	0.00	0.01	0.00	0.00	0.02	0.00	0.39	0.01	0.00	0.00	0.00	0.00	0.00	0.00
Kitere	Ke	Uganda	0.00	0.04	0.03	0.00	0.71	0.11	0.00	0.00	0.00	0.00	0.07	0.01	0.00	0.00	0.00	0.01	0.00	0.00	0.00	0.00	0.00	0.00	0.00	0.00	0.00	0.00	0.00	0.00	0.00	0.00	0.00	0.00	0.00	0.00
Kyanga	Ky	Uganda	0.00	0.00	0.02	0.00	0.40	0.05	0.00	0.00	0.02	0.00	0.39	0.05	0.00	0.00	0.00	0.00	0.01	0.00	0.00	0.00	0.00	0.01	0.00	0.00	0.00	0.00	0.01	0.00	0.00	0.00	0.00	0.01	0.00	0.00
Kyaninga	Kg	Uganda	0.00	0.00	0.01	0.00	0.10	0.02	0.00	0.00	0.00	0.02	0.11	0.06	0.00	0.00	0.00	0.09	0.05	0.02	0.00	0.00	0.00	0.01	0.01	0.05	0.06	0.00	0.13	0.09	0.01	0.00	0.04	0.05	0.05	0.00
Large Hall Tarn	Lht	Kenya	0.00	0.00	0.01	0.06	0.24	0.15	0.00	0.00	0.00	0.03	0.03	0.02	0.00	0.01	0.00	0.00	0.02	0.01	0.01	0.00	0.00	0.00	0.00	0.00	0.30	0.00	0.10	0.01	0.00	0.00	0.00	0.00	0.00	0.00
Lower Kachope	Lka	Uganda	0.00	0.00	0.02	0.10	0.25	0.04	0.00	0.00	0.00	0.03	0.07	0.05	0.00	0.07	0.00	0.04	0.06	0.04	0.00	0.00	0.00	0.00	0.00	0.00	0.09	0.00	0.10	0.04	0.00	0.00	0.00	0.00	0.00	0.00
Lower Kitandara	Lki	Uganda	0.00	0.00	0.02	0.11	0.21	0.05	0.00	0.00	0.00	0.01	0.02	0.02	0.01	0.15	0.00	0.00	0.07	0.07	0.00	0.00	0.00	0.00	0.00	0.00	0.14	0.00	0.09	0.04	0.00	0.00	0.00	0.00	0.00	0.00
Mahuhura	Ma	Uganda	0.00	0.00	0.05	0.01	0.60	0.08	0.00	0.00	0.01	0.01	0.18	0.04	0.00	0.00	0.00	0.00	0.01	0.00	0.00	0.00	0.00	0.00	0.00	0.00	0.00	0.00	0.01	0.00	0.00	0.00	0.00	0.00	0.00	0.00
Mbajo	Mb	Uganda	0.00	0.00	0.03	0.00	0.61	0.07	0.00	0.00	0.01	0.00	0.20	0.02	0.00	0.00	0.00	0.01	0.00	0.00	0.00	0.00	0.00	0.01	0.00	0.00	0.00	0.00	0.01	0.00	0.00	0.01	0.00	0.00	0.00	0.00
Middle Kachope	Mka	Uganda	0.00	0.00	0.03	0.12	0.27	0.07	0.00	0.00	0.01	0.02	0.07	0.04	0.00	0.06	0.00	0.04	0.05	0.02	0.00	0.00	0.00	0.00	0.00	0.00	0.07	0.00	0.06	0.02	0.05	0.00	0.00	0.00	0.00	0.00
Murabio	Mu	Uganda	0.00	0.00	0.03	0.00	0.69	0.07	0.00	0.00	0.00	0.00	0.11	0.01	0.00	0.00	0.00	0.01	0.01	0.02	0.01	0.00	0.00	0.00	0.00	0.00	0.00	0.00	0.01	0.00	0.00	0.00	0.01	0.01	0.00	0.00
Murusi	Mr	Uganda	0.00	0.00	0.03	0.00	0.76	0.09	0.00	0.00	0.01	0.00	0.06	0.01	0.00	0.00	0.00	0.00	0.01	0.00	0.00	0.00	0.00	0.00	0.00	0.00	0.00	0.00	0.01	0.00	0.00	0.00	0.00	0.01	0.00	0.00
Mutinda	Mt	Uganda	0.00	0.01	0.05	0.16	0.34	0.10	0.00	0.00	0.01	0.02	0.03	0.04	0.00	0.10	0.01	0.01	0.04	0.05	0.00	0.00	0.00	0.00	0.00	0.00	0.00	0.00	0.02	0.00	0.00	0.00	0.00	0.00	0.00	0.00
Njarayabana	Nj	Uganda	0.00	0.00	0.05	0.00	0.73	0.10	0.00	0.00	0.01	0.00	0.07	0.01	0.00	0.00	0.00	0.00	0.01	0.00	0.00	0.00	0.00	0.00	0.00	0.00	0.00	0.00	0.00	0.00	0.00	0.00	0.00	0.00	0.00	0.00
Nyamswiga	Ny	Uganda	0.00	0.00	0.01	0.00	0.20	0.03	0.00	0.00	0.02	0.02	0.43	0.04	0.00	0.00	0.00	0.00	0.01	0.00	0.00	0.00	0.00	0.01	0.00	0.00	0.01	0.00	0.17	0.01	0.01	0.00	0.01	0.01	0.00	0.00
Nyamugosani	Na	Uganda	0.00	0.00	0.03	0.01	0.63	0.07	0.00	0.00	0.01	0.01	0.19	0.03	0.00	0.00	0.00	0.00	0.00	0.00	0.00	0.00	0.00	0.00	0.00	0.00	0.00	0.00	0.00	0.00	0.00	0.00	0.00	0.00	0.00	0.00
Nyantonde	Nt	Uganda	0.00	0.00	0.03	0.00	0.45	0.05	0.00	0.00	0.01	0.00	0.32	0.04	0.00	0.00	0.00	0.01	0.00	0.00	0.00	0.00	0.00	0.02	0.00	0.00	0.00	0.00	0.01	0.01	0.00	0.00	0.01	0.01	0.00	0.00
Nyungu	Nu	Uganda	0.00	0.00	0.04	0.00	0.67	0.10	0.00	0.00	0.01	0.00	0.15	0.02	0.00	0.00	0.00	0.00	0.01	0.00	0.00	0.00	0.00	0.01	0.00	0.00	0.00	0.00	0.00	0.00	0.00	0.00	0.00	0.00	0.00	0.00
Oblong Tarn	Ot	Kenya	0.00	0.00	0.01	0.03	0.12	0.01	0.00	0.00	0.00	0.00	0.01	0.03	0.00	0.06	0.00	0.04	0.03	0.00	0.00	0.00	0.00	0.00	0.00	0.00	0.40	0.00	0.20	0.06	0.00	0.00	0.00	0.00	0.00	0.00
Saka	Sk	Uganda	0.00	0.00	0.02	0.00	0.39	0.02	0.00	0.00	0.01	0.02	0.26	0.02	0.00	0.00	0.00	0.00	0.01	0.00	0.00	0.00	0.00	0.01	0.00	0.00	0.01	0.00	0.16	0.02	0.00	0.00	0.01	0.02	0.01	0.00
Speke	Sp	Democractic Republic of the Congo	0.00	0.00	0.01	0.17	0.28	0.04	0.00	0.00	0.00	0.06	0.06	0.09	0.00	0.11	0.00	0.03	0.05	0.00	0.00	0.00	0.00	0.00	0.00	0.00	0.05	0.00	0.04	0.01	0.00	0.00	0.00	0.00	0.00	0.00
Tanganyika	Ta	Tanzania	0.00	0.00	0.01	0.00	0.79	0.07	0.00	0.00	0.00	0.00	0.04	0.01	0.00	0.00	0.00	0.00	0.01	0.00	0.01	0.00	0.00	0.00	0.00	0.00	0.00	0.00	0.03	0.01	0.00	0.00	0.00	0.00	0.00	0.00
Upper Kachope	Uka	Uganda	0.00	0.01	0.04	0.13	0.31	0.12	0.00	0.00	0.01	0.03	0.03	0.08	0.00	0.04	0.00	0.02	0.06	0.00	0.00	0.00	0.00	0.00	0.00	0.00	0.06	0.00	0.05	0.01	0.00	0.00	0.00	0.00	0.00	0.00
Upper Kitandara	Uki	Uganda	0.00	0.00	0.01	0.06	0.13	0.03	0.00	0.00	0.00	0.01	0.01	0.02	0.00	0.21	0.00	0.11	0.14	0.00	0.00	0.00	0.00	0.00	0.00	0.00	0.21	0.00	0.05	0.01	0.00	0.00	0.00	0.00	0.00	0.00
Other lakes and ponds																																				
Conophyton Pond	CP	Antarctica	0.00	0.00	0.20	0.20	0.24	0.08	0.07	0.06	0.00	0.00	0.00	0.00	0.00	0.11	0.00	0.00	0.04	0.00	0.00	0.00	0.00	0.00	0.00	0.00	0.00	0.00	0.00	0.00	0.00	0.00	0.00	0.00	0.00	0.00
Orange Pond	OP	Antarctica	0.00	0.00	0.24	0.19	0.26	0.09	0.12	0.06	0.00	0.00	0.00	0.00	0.00	0.02	0.00	0.00	0.01	0.00	0.00	0.00	0.00	0.00	0.00	0.00	0.00	0.00	0.00	0.00	0.00	0.00	0.00	0.00	0.00	0.00
Laguna Potrok Aike	Pa	Argentina	0.00	0.00	0.00	0.03	0.05	0.02	0.00	0.00	0.00	0.00	0.03	0.00	0.00	0.00	0.00	0.00	0.00	0.00	0.00	0.00	0.00	0.00	0.00	0.06	0.00	0.00	0.05	0.66	0.00	0.00	0.00	0.07	0.00	0.00
Lake Constance	Co	Germany	0.00	0.01	0.01	0.07	0.25	0.05	0.00	0.00	0.01	0.05	0.03	0.07	0.08	0.01	0.00	0.33	0.00	0.00	0.00	0.00	0.00	0.00	0.00	0.01	0.00	0.00	0.02	0.02	0.00	0.00	0.00	0.00	0.00	0.00
Lake Schreventeich[1]	St	Germany	0.00	0.00	0.00	0.16	0.59	0.00	0.00	0.00	0.00	0.06	0.09	0.00	0.00	0.04	0.00	0.07	0.00	0.00	0.00	0.00	0.00	0.00	0.00	0.00	0.00	0.00	0.00	0.00	0.00	0.00	0.00	0.00	0.00	0.00
Lake Lading	La	Indonesia	0.00	0.00	0.00	0.00	0.08	0.01	0.00	0.00	0.00	0.00	0.03	0.03	0.00	0.00	0.00	0.00	0.00	0.01	0.00	0.00	0.00	0.00	0.02	0.01	0.02	0.00	0.11	0.09	0.18	0.06	0.05	0.19	0.03	0.07
Lake Klakah	Kl	Indonesia	0.00	0.00	0.00	0.00	0.73	0.13	0.00	0.00	0.00	0.00	0.05	0.01	0.00	0.00	0.00	0.02	0.00	0.00	0.00	0.00	0.00	0.00	0.00	0.00	0.00	0.00	0.03	0.00	0.00	0.01	0.00	0.00	0.00	0.00
Lake Towuti	To	Indonesia	0.00	0.00	0.00	0.00	0.08	0.00	0.00	0.00	0.05	0.00	0.37	0.09	0.00	0.00	0.00	0.03	0.07	0.21	0.00	0.01	0.00	0.01	0.02	0.00	0.00	0.00	0.00	0.00	0.00	0.01	0.01	0.03	0.00	0.00

[1]Bauersachs et al. (2015)

Supplementary Table 3. Fractional abundances of HG_{26} diols and HG_{26} keto-ols, HDI_{26} values, HDI_{26}-calculated surface water temperatures (SWT) and residuals from surface sediments of East African lakes as well as lakes and ponds from polar to tropical latitudes.

Lake	Abbreviation	Country	HG_{26} keto-ols				HG_{26} diols				HDI_{26}	STD HDI_{26}	HDI_{26}-SWT	Residuals
			I	STD I	II	STD II	I	STD I	II	STD II			(°C)	(°C)
East African lakes														
Albert	Al	Uganda	0.013	0.001	0.002	0.000	0.964	0.016	0.021	0.017	0.985	0.001	27.32	0.46
Bandasa	Ba	Kenya	0.030		0.154		0.670		0.145		0.813		16.20	1.00
Bigata	Bi	Uganda	0.053		0.249		0.561		0.136		0.692		8.42	0.58
Bugwagi	Bu	Uganda	0.031		0.008		0.846		0.115		0.965		25.98	0.52
Bujuku	Bk	Uganda	0.036		0.254		0.545		0.165		0.683		7.79	0.61
Bukurungu East	Be	Uganda	0.048	0.003	0.230	0.008	0.539	0.003	0.182	0.033	0.701	0.002	8.95	0.75
Edward	Ed	Uganda	0.032		0.003		0.842		0.123		0.963		25.89	0.91
Eldoret Nakuru 1a	El 1	Kenya	0.091		0.146		0.670		0.092		0.821		16.69	-2.59
Eldoret Nakuru 2	El 2	Kenya	0.022		0.102		0.647		0.229		0.863		19.43	-4.94
Eldoret Nakuru 3	El 3	Kenya	0.031	0.008	0.133	0.013	0.790	0.018	0.046	0.003	0.856	0.015	18.97	-2.67
Ellis	Es	Kenya	0.014		0.187		0.726		0.073		0.795		15.06	-2.46
Enchanted	En	Kenya	0.019		0.305		0.571		0.105		0.652		5.80	2.40
Hausburg Tarn	Hat	Kenya	0.019		0.131		0.650		0.200		0.832		17.45	-9.55
Hohnell	Ho	Kenya	0.021		0.392		0.562		0.025		0.589		1.77	6.13
Hut Tarn	Hut	Kenya	0.025		0.184		0.672		0.118		0.785		14.41	-8.71
Ibanda Pool	Ib	Uganda	0.033	0.010	0.194	0.001	0.720	0.011	0.053	0.001	0.787	0.004	14.54	0.76
Karolero	Ka	Uganda	0.024		0.009		0.843		0.124		0.972		26.49	0.21
Kasenda	Ks	Uganda	0.035		0.007		0.857		0.101		0.961		25.73	0.07
Kasirya	Kr	Uganda	0.032		0.005		0.864		0.099		0.964		25.95	0.35
Katalin	Kt	Kenya	0.047		0.129		0.649		0.174		0.834		17.54	1.96
Kibengo	Kb	Uganda	0.008		0.003		0.899		0.090		0.991		27.67	0.03
Kifuruka	Ki	Uganda	0.053		0.008		0.808		0.131		0.939		24.32	-0.92
Kisibendi	Kn	Uganda	0.049	0.001	0.020	0.002	0.842	0.002	0.089	0.005	0.945	0.001	24.73	0.27
Kitere	Ke	Uganda	0.033		0.004		0.829		0.134		0.962		25.80	0.90
Kyanga	Ky	Uganda	0.043	0.012	0.005	0.005	0.841	0.020	0.111	0.037	0.952	0.002	25.14	1.76
Kyaninga	Kg	Uganda	0.062		0.008		0.809		0.121		0.929		23.67	0.13
Large Hall Tarn	Lht	Kenya	0.014		0.135		0.523		0.328		0.795		15.01	-6.31
Lower Kachope	Lka	Uganda	0.057		0.240		0.605		0.098		0.716		9.97	-1.57
Lower Kitandara	Lki	Uganda	0.043		0.282		0.546		0.129		0.659		6.28	0.22
Mahuhura	Ma	Uganda	0.065		0.011		0.821		0.104		0.927		23.55	1.05
Mbajo	Mb	Uganda	0.047		0.007		0.850		0.096		0.947		24.87	-0.37
Middle Kachope	Mka	Uganda	0.064		0.243		0.556		0.137		0.696		8.67	0.03
Murabio	Mu	Uganda	0.036	0.001	0.006	0.001	0.876	0.007	0.082	0.008	0.960	0.000	25.70	0.99
Murusi	Mr	Uganda	0.038		0.005		0.860		0.097		0.958		25.55	0.55
Mutinda	Mt	Uganda	0.069	0.002	0.252	0.007	0.523	0.004	0.155	0.009	0.675	0.005	7.29	0.41
Njarayabana	Nj	Uganda	0.056		0.005		0.822		0.117		0.936		24.16	1.34
Nyamswiga	Ny	Uganda	0.002	0.002	0.006	0.000	0.841	0.001	0.121	0.001	0.963	0.002	25.88	-2.29
Nyamugosani	Na	Uganda	0.044		0.012		0.846		0.098		0.951		25.08	1.72
Nyantonde	Nt	Uganda	0.051		0.005		0.856		0.089		0.944		24.66	-0.96
Nyungu	Nu	Uganda	0.044		0.003		0.832		0.120		0.949		25.00	-0.30
Oblong Tarn	Ot	Kenya	0.037		0.185		0.704		0.074		0.792		14.84	-6.44
Saka	Sk	Uganda	0.036		0.005		0.921		0.038		0.963		25.85	-3.15
Speke	Sp	Democractic Republic of the Congo	0.018	0.001	0.337	0.006	0.566	0.000	0.079	0.007	0.627	0.004	4.19	1.61
Tanganyika	Ta	Tanzania	0.015		0.003		0.897		0.085		0.984		27.22	-1.52
Upper Kachope	Uka	Uganda	0.068		0.214		0.517		0.201		0.707		9.38	-1.58
Upper Kitandara	Uki	Uganda	0.030		0.279		0.575		0.117		0.673		7.20	-1.40
Average														1.82
Other lakes and ponds														
Conophyton Pond	CP	Antarctica	0.277		0.271		0.337		0.116		0.549		2.70	-1.70
Orange Pond	OP	Antarctica	0.313		0.248		0.330		0.109		0.513		0.52	1.98
Laguna Potrok Aike	Pa	Argentina	0.016		0.268		0.519		0.196		0.660	0.002	9.31	0.14
Lake Constance	Co	Germany	0.009		0.163		0.760		0.068		0.823		19.11	1.46
Lake Schreventeich[1]	St	Germany	0.000		0.209		0.791		0.000		0.791		17.17	-1.47
Lake Lading	La	Indonesia	0.008		0.006		0.871		0.114		0.991		29.15	-0.44
Lake Klakah	Kl	Indonesia	0.002		0.001		0.847		0.150		0.997		29.54	-0.04
Lake Towuti	To	Indonesia	0.000		0.000		0.945		0.055		1.000	0.001	29.69	0.11
Average														1.68

[1]Bauersachs et al. (2015)

Supplementary Table 4. Fractional abundances of heterocyte glycolipids (HG) detected in the sediment record of Lake Tanganyika, Tanzania.

Core ID	Section	Top Depth	Bottom Depth	Depth	Age	DE HG_{26} diol	P HG_{26} diol	HG_{26} keto-ols		HG_{26} diols		HG_{28} keto-ols		HG_{28} diols		HG_{28} keto-diols		HG_{28} triols			HG_{30} keto-ols		HG_{30} diols		HG_{30} keto-diols			HG_{30} triols			HG_{32} keto-diols		HG_{32} triols	
		(cm)	(cm)	(cm)	(years BP)	I	I	I	II	I	II	I	II	I	II	I	II	I	II	III	I	II	I	II	I	II	III	I	II	III	I	II	I	II
NP04-KH-4A-1K	1	20	21	12.0	294	0.00	0.00	0.01	0.00	0.47	0.00	0.02	0.00	0.29	0.01	0.00	0.00	0.01	0.00	0.00	0.00	0.00	0.00	0.00	0.00	0.00	0.00	0.16	0.01	0.00	0.00	0.00	0.00	0.00
NP04-KH-4A-1K	1	48	49	40.0	980	0.00	0.00	0.00	0.00	0.02	0.00	0.02	0.01	0.49	0.03	0.00	0.00	0.00	0.01	0.00	0.00	0.00	0.00	0.01	0.01	0.00	0.00	0.36	0.01	0.00	0.00	0.00	0.02	0.00
NP04-KH-4A-1K	1	80	81	72.0	1764	0.00	0.00	0.00	0.00	0.00	0.00	0.01	0.01	0.63	0.01	0.00	0.00	0.00	0.00	0.00	0.00	0.00	0.00	0.01	0.00	0.01	0.00	0.31	0.00	0.00	0.00	0.00	0.00	0.00
NP04-KH-4A-1K	1	110	111	102.0	2499	0.00	0.00	0.00	0.00	0.05	0.00	0.03	0.01	0.61	0.01	0.00	0.00	0.00	0.00	0.00	0.00	0.00	0.00	0.01	0.00	0.01	0.00	0.26	0.00	0.00	0.00	0.00	0.00	0.00
NP04-KH-4A-1K	1	140	141	132.0	3234	0.00	0.00	0.00	0.00	0.05	0.00	0.01	0.02	0.54	0.00	0.00	0.00	0.00	0.00	0.00	0.00	0.00	0.00	0.01	0.00	0.01	0.00	0.32	0.01	0.00	0.00	0.00	0.01	0.00
NP04-KH-4A-1K	2	20	21	171.5	4201	0.00	0.00	0.00	0.00	0.01	0.00	0.03	0.02	0.50	0.02	0.00	0.00	0.00	0.00	0.00	0.00	0.00	0.00	0.01	0.00	0.02	0.00	0.34	0.02	0.00	0.00	0.00	0.02	0.00
NP04-KH-4A-1K	2	80	81	231.5	6885	0.00	0.00	0.01	0.01	0.16	0.01	0.03	0.00	0.34	0.06	0.00	0.00	0.00	0.00	0.00	0.00	0.00	0.00	0.00	0.01	0.00	0.00	0.26	0.05	0.00	0.00	0.00	0.07	0.00
NP04-KH-4A-1K	2	110	111	261.5	8346	0.00	0.00	0.02	0.01	0.24	0.02	0.02	0.00	0.18	0.14	0.00	0.00	0.03	0.00	0.00	0.00	0.00	0.00	0.00	0.02	0.00	0.00	0.07	0.08	0.00	0.03	0.00	0.15	0.00
NP04-KH-4A-1K	2	140	141	291.5	9807	0.00	0.00	0.02	0.01	0.33	0.00	0.00	0.01	0.14	0.10	0.00	0.00	0.04	0.00	0.00	0.00	0.00	0.00	0.00	0.00	0.00	0.00	0.08	0.16	0.00	0.00	0.00	0.11	0.00
NP04-KH-4A-1K	3	22	23	323.5	11365	0.00	0.00	0.00	0.00	0.01	0.00	0.01	0.04	0.31	0.02	0.00	0.00	0.00	0.00	0.00	0.00	0.00	0.00	0.01	0.03	0.00	0.00	0.32	0.23	0.00	0.00	0.00	0.01	0.00
NP04-KH-4A-1K	3	52	53	353.5	12826	0.00	0.00	0.02	0.01	0.22	0.01	0.02	0.03	0.20	0.01	0.00	0.00	0.01	0.02	0.00	0.00	0.00	0.00	0.00	0.04	0.00	0.00	0.18	0.22	0.00	0.00	0.00	0.03	0.00
NP04-KH-4A-1K	3	80	81	381.5	14190	0.00	0.00	0.00	0.00	0.03	0.00	0.02	0.06	0.49	0.02	0.00	0.00	0.00	0.00	0.00	0.00	0.00	0.00	0.01	0.03	0.00	0.00	0.23	0.09	0.00	0.00	0.00	0.01	0.00
NP04-KH-4A-1K	3	110	111	411.5	14980	0.00	0.01	0.00	0.00	0.02	0.00	0.01	0.01	0.05	0.02	0.00	0.00	0.01	0.01	0.00	0.00	0.00	0.00	0.00	0.07	0.00	0.00	0.27	0.41	0.00	0.01	0.01	0.08	0.01
NP04-KH-4A-1K	4	13	14	445.0	18923	0.00	0.00	0.01	0.00	0.09	0.00	0.02	0.05	0.13	0.32	0.00	0.00	0.00	0.00	0.00	0.00	0.00	0.00	0.00	0.03	0.00	0.00	0.10	0.13	0.00	0.00	0.00	0.12	0.00
NP04-KH-4A-1K	4	40	41	472.0	21766	0.00	0.00	0.00	0.00	0.03	0.00	0.00	0.02	0.03	0.17	0.00	0.00	0.03	0.00	0.00	0.00	0.00	0.00	0.00	0.13	0.00	0.00	0.22	0.33	0.00	0.00	0.00	0.02	0.00
NP04-KH-4A-1K	4	68	69	500.0	25230	0.00	0.00	0.00	0.00	0.04	0.00	0.03	0.02	0.11	0.12	0.01	0.00	0.02	0.00	0.00	0.00	0.00	0.00	0.00	0.02	0.00	0.00	0.14	0.40	0.00	0.04	0.00	0.03	0.00
NP04-KH-4A-1K	4	101	102	533.0	26721	0.00	0.00	0.00	0.00	0.00	0.00	0.00	0.00	0.01	0.01	0.00	0.00	0.01	0.00	0.00	0.00	0.00	0.00	0.00	0.02	0.00	0.00	0.33	0.61	0.00	0.00	0.00	0.01	0.00
NP04-KH-4A-1K	4	128	129	560.0	28157	0.00	0.00	0.01	0.00	0.05	0.00	0.04	0.01	0.02	0.12	0.00	0.00	0.00	0.00	0.00	0.00	0.00	0.00	0.00	0.00	0.00	0.00	0.17	0.49	0.04	0.02	0.00	0.02	0.00
NP04-KH-4A-1K	5	8	9	590.5	30417	0.00	0.00	0.00	0.00	0.02	0.00	0.00	0.00	0.01	0.02	0.00	0.00	0.00	0.00	0.00	0.00	0.00	0.00	0.00	0.02	0.01	0.00	0.21	0.70	0.00	0.00	0.00	0.01	0.00
NP04-KH-4A-1K	5	38	39	620.5	32049	0.00	0.00	0.00	0.00	0.02	0.00	0.00	0.00	0.01	0.02	0.00	0.00	0.01	0.00	0.00	0.00	0.00	0.02	0.00	0.05	0.00	0.00	0.28	0.57	0.00	0.00	0.00	0.00	0.00
NP04-KH-4A-1K	5	70	71	652.5	33946	0.00	0.00	0.00	0.00	0.01	0.00	0.01	0.00	0.01	0.03	0.00	0.00	0.00	0.00	0.00	0.00	0.00	0.00	0.00	0.01	0.00	0.00	0.18	0.74	0.00	0.00	0.00	0.01	0.00
NP04-KH-4A-1K	5	101	102	683.5	35784	0.00	0.00	0.00	0.00	0.01	0.00	0.01	0.01	0.05	0.02	0.00	0.01	0.01	0.03	0.00	0.00	0.00	0.00	0.00	0.01	0.01	0.00	0.26	0.54	0.00	0.01	0.00	0.02	0.00
NP04-KH-4A-1K	5	120	121	702.5	36911	0.00	0.01	0.00	0.00	0.06	0.01	0.02	0.02	0.25	0.07	0.00	0.01	0.02	0.00	0.00	0.00	0.00	0.00	0.00	0.00	0.03	0.00	0.18	0.32	0.00	0.00	0.00	0.00	0.00

Supplementary Table 5. Fractional abundances of HG_{26} keto-ols and HG_{26} diols, HDI_{26} values and HDI_{26}-calculated surface water temperatures (SWT) in the sediment record of Lake Tanganyika, Tanzania.

Core ID	Section	Top Depth	Bottom Depth	Depth	Age	HG_{26} keto-ols		HG_{26} diols		HDI_{26}	STD HDI_{26}	HDI_{26}-SWT	STD HDI_{26}-SWT
		(cm)	(cm)	(cm)	(years BP)	I	II	I	II			(°C)	(°C)
NP04-KH-4A-1K	1	20	21	12.0	294	0.03	0.00	0.96	0.01	0.972	0.005	26.49	0.32
NP04-KH-4A-1K	1	48	49	40.0	980	0.05	0.04	0.87	0.05	0.947	0.001	24.83	0.06
NP04-KH-4A-1K	1	80	81	72.0	1764	0.04	0.03	0.71	0.23	0.944	0.001	24.68	0.08
NP04-KH-4A-1K	1	110	111	102.0	2499	0.04	0.01	0.93	0.03	0.959	0.002	25.62	0.14
NP04-KH-4A-1K	1	140	141	132.0	3234	0.04	0.02	0.89	0.05	0.960	0.002	25.69	0.10
NP04-KH-4A-1K	2	20	21	171.5	4201	0.03	0.03	0.90	0.04	0.973	0.000	26.50	0.02
NP04-KH-4A-1K	2	80	81	231.5	6885	0.05	0.04	0.87	0.03	0.945	0.005	24.71	0.31
NP04-KH-4A-1K	2	110	111	261.5	8346	0.05	0.03	0.86	0.06	0.940	0.004	24.38	0.24
NP04-KH-4A-1K	2	140	141	291.5	9807	0.04	0.04	0.92	0.00	0.954	0.002	25.42	0.15
NP04-KH-4A-1K	3	22	23	323.5	11365	0.05	0.00	0.80	0.15	0.941	0.000	24.46	0.01
NP04-KH-4A-1K	3	52	53	353.5	12826	0.07	0.02	0.87	0.04	0.922	0.000	23.24	0.03
NP04-KH-4A-1K	3	80	81	381.5	14190	0.06	0.03	0.83	0.08	0.928	0.002	23.62	0.10
NP04-KH-4A-1K	3	110	111	411.5	14980	0.07	0.02	0.91	0.00	0.929	0.001	23.68	0.08
NP04-KH-4A-1K	4	13	14	445.0	18923	0.09	0.00	0.91	0.00	0.913	0.002	22.55	0.13
NP04-KH-4A-1K	4	40	41	472.0	21766	0.09	0.00	0.91	0.00	0.909	0.001	22.37	0.05
NP04-KH-4A-1K	4	68	69	500.0	25230	0.09	0.00	0.91	0.00	0.908	0.005	22.35	0.31
NP04-KH-4A-1K	4	101	102	533.0	26721	0.07	0.02	0.67	0.24	0.910	0.003	22.49	0.17
NP04-KH-4A-1K	4	128	129	560.0	28157	0.09	0.00	0.91	0.00	0.911	0.003	22.52	0.19
NP04-KH-4A-1K	5	8	9	590.5	30417	0.08	0.00	0.92	0.00	0.917	0.004	22.88	0.23
NP04-KH-4A-1K	5	38	39	620.5	32049	0.08	0.00	0.92	0.00	0.922	0.001	23.21	0.07
NP04-KH-4A-1K	5	70	71	652.5	33946	0.06	0.00	0.75	0.18	0.922	0.001	23.21	0.04
NP04-KH-4A-1K	5	101	102	683.5	35784	0.05	0.00	0.63	0.31	0.923	0.004	23.30	0.24
NP04-KH-4A-1K	5	120	121	702.5	36911	0.06	0.00	0.80	0.14	0.932	0.002	23.85	0.14

Supplementary Table 6. Bivariate correlation analysis of environmental parameters and heterocyte glycolipids (HG) with the HDI_{26} in surface sediments of tropical East African lakes.

		Lake Depth	Lake Surface Area	MAAT	SWT	BWT	SW pH	BW pH	SW DO	BW DO	Conductivity	TP	TN	DOC	Chl a	TOC	TON	HG_{26} keto-ol (I)	HG_{26} keto-ol (II)	Combined HG_{26} keto-ols (I + II)	HG_{26} diol (I)	HG_{26} diol (II)	HDI_{26}
Elevation	r	-0,410**	-0.220	-0,992**	-0,972**	-0,975**	-0,778**	-0,608**	0.261	0,656**	-0,770**	-0.304	-0.091	0.242	-0.142	0.243	0.052	0.141	0,960**	0,960**	-0,947**	0.274	-0,973**
	p	0.007	0.162	0.000	0.000	0.000	0.000	0.000	0.104	0.000	0.000	0.064	0.585	0.245	0.551	0.131	0.811	0.375	0.000	0.000	0.000	0.080	0.000
	n	42	42	42	42	42	42	42	40	41	42	42	38	25	20	40	24	42	42	42	42	42	42
Lake Depth	r		0,385*	0,403**	0,431**	0,407**	0,441**	0.226	-0.01	-0,356*	0,466**	-0.057	-0,329*	-0.366	-0.211	-0.136	-0.235	-0.083	-0,435**	0,401**	0,448**	-0.181	0,409**
	p		0.012	0.008	0.004	0.007	0.003	0.150	0.953	0.023	0.002	0.733	0.044	0.072	0.372	0.404	0.270	0.600	0.004	0.008	0.003	0.252	0.007
	n		42	42	42	42	42	42	40	41	42	38	38	25	20	40	24	42	42	42	42	42	42
Lake Surface Area	r			0.194	0.175	0.198	0.198	0,351*	0.103	0.011	0.241	-0.087	-0.166	0.077	-0.145	-0.185	-0.228	-0.298	-0.169	-0.200	0.228	-0.164	0.193
	p			0.219	0.268	0.209	0.209	0.023	0.526	0.944	0.125	0.604	0.318	0.715	0.541	0.253	0.284	0.055	0.284	0.205	0.146	0.301	0.220
	n			42	42	42	42	42	40	41	42	38	38	25	20	40	24	42	42	42	42	42	42
MAAT	r				0,979**	0,984**	0,809**	0,625**	-0.275	-0,644**	0,772**	0.315	0.107	-0.240	0.187	-0.270	-0.093	-0.200	-0,948**	-0,947**	0,956**	-0,311*	0,970**
	p				0.000	0.000	0.000	0.000	0.085	0.000	0.000	0.054	0.523	0.248	0.430	0.092	0.665	0.205	0.000	0.000	0.000	0.045	0.000
	n				42	42	42	42	40	41	42	38	38	25	20	40	24	42	42	42	42	42	42
SWT	r					0,991**	0,794**	0,578**	-0.265	-0,739**	0,786**	0,329*	0.119	-0.195	0.192	-0.193	-0.056	-0.181	-0,964**	-0,979**	0,953**	-0.256	0,975**
	p					0.000	0.000	0.000	0.099	0.000	0.000	0.043	0.478	0.351	0.417	0.232	0.795	0.252	0.000	0.000	0.000	0.102	0.000
	n					42	42	42	40	41	42	38	38	25	20	40	24	42	42	42	42	42	42
BWT	r						0,809**	0,633**	-0.288	-0,682**	0,769**	0,344*	0.141	-0.180	0.235	-0.268	-0.109	-0.206	-0,951**	-0,951**	0,948**	-0.275	0,969**
	p						0.000	0.000	0.072	0.000	0.000	0.034	0.398	0.389	0.318	0.095	0.613	0.191	0.000	0.000	0.000	0.078	0.000
	n						42	42	40	41	42	38	38	25	20	40	24	42	42	42	42	42	42
SW pH	r							0,741**	-0.153	-0,332*	0,672**	0,343*	0.207	-0.150	0.153	-0.300	-0.204	-0.271	-0,737**	-0,722**	0,793**	-0,363*	0,758**
	p							0.000	0.344	0.034	0.000	0.035	0.212	0.476	0.520	0.060	0.340	0.083	0.000	0.000	0.000	0.018	0.000
	n							42	40	41	42	38	38	25	20	40	24	42	42	42	42	42	42
BW pH	r								-0.206	-0.043	0,513**	0,378*	0.194	0.091	0.315	-0,455**	-0,449*	-0,351*	0,537**	-0,563**	0,594**	-0.279	0,576**
	p								0.203	0.790	0.001	0.019	0.244	0.664	0.176	0.003	0.028	0.023	0.000	0.000	0.000	0.073	0.000
	n								40	41	42	38	38	25	20	40	24	42	42	42	42	42	42
SW DO	r									0.300	-0.090	-0.298	-0.234	-0.144	-0,786**	0.138	-0.059	-0.046	0.279	0.293	-0.208	-0.143	-0.292
	p									0.060	0.582	0.077	0.169	0.503	0.000	0.410	0.788	0.778	0.081	0.067	0.198	0.377	0.068
	n									40	40	36	36	24	19	38	23	40	40	40	40	40	40
BW DO	r										-0,577**	-0.296	-0.118	0.100	-0.140	-0.129	-0.252	-0.153	0,752**	0,727**	-0,642**	-0.064	-0,718**
	p										0.000	0.075	0.487	0.643	0.568	0.434	0.234	0.341	0.000	0.000	0.000	0.689	0.000
	n										41	37	37	24	19	39	24	41	41	41	41	41	41
Conductivity	r											0,398*	-0.184	-0.347	-0.073	-0.150	-0.177	-0.058	-0,761**	-0,733**	0,764**	-0.269	0,751**
	p											0.013	0.269	0.089	0.760	0.356	0.407	0.715	0.000	0.000	0.000	0.085	0.000
	n											38	38	25	20	40	24	42	42	42	42	42	42
TP	r												0,447**	0.099	0,938**	-0.296	-0.211	0.142	-0.305	-0.285	0.248	0.044	0.293
	p												0.005	0.636	0.000	0.080	0.322	0.394	0.063	0.083	0.133	0.794	0.740
	n												38	25	19	36	24	38	38	38	38	38	38
TN	r													0,680**	0,898**	-0.058	0.128	-0.023	-0.117	-0.133	0.083	0.090	0.127
	p													0.000	0.000	0.739	0.551	0.890	0.486	0.428	0.618	0.590	0.448
	n													25	19	36	24	38	38	38	38	38	38
DOC	r														0,552**	-0.085	-0.062	-0.003	0.182	0.132	-0.241	0.277	-0.175
	p														0.033	0.692	0.819	0.989	0.384	0.528	0.245	0.180	0.402
	n														15	24	16	25	25	25	25	25	25
Chl a	r															-0.395	-0,584*	0.073	-0.133	-0.162	0.095	0.051	0.164
	p															0.084	0.046	0.760	0.576	0.496	0.689	0.830	0.489
	n															20	12	20	20	20	20	20	20
TOC	r																0,837**	0.235	0.106	0.141	-0.148	0.119	-0.158
	p																0.000	0.144	0.513	0.385	0.363	0.464	0.330
	n																24	40	40	40	40	40	40
TON	r																	0.386	-0.053	-0.038	-0.068	0.288	0.015
	p																	0.063	0.806	0.858	0.752	0.172	0.946
	n																	24	24	24	24	24	24
HG_{26} keto-ol (I)	r																		0.062	0.104	-0.282	0.333	-0.155
	p																		0.696	0.513	0.071	0.031	0.327
	n																		42	42	42	42	42
HG_{26} keto-ol (II)	r																			0,994**	-0,929**	0.123	-0,990**
	p																			0.000	0.000	0.438	0.000
	n																			42	42	42	42
Combined HG_{26} keto-ols (I + II)	r																				-0,924**	0.110	-0,992**
	p																				0.000	0.490	0.000
	n																				42	42	42
HG_{26} diol (I)	r																					-0,460**	0,960**
	p																					0.002	0.000
	n																					42	42
HG_{26} diol (II)	r																						-0.213
	p																						0.176
	n																						42

* Correlation is significant at 0.05 level
** Correlation is significant at 0.01 level

Supplementary Table 7. Partial correlation analysis of environmental parameters and heterocyte glycolipids (HG) with the HDI_{26} in surface sediments of tropical East African lakes after removing the effect of surface water temperature (SWT).

Control Variable *SWT*		Lake Depth	Lake Surface Area	MAAT	BWT	SW pH	BW pH	SW DO	BW DO	Conductivity	TP	TN	DOC	Chl a	TOC	TON	HG_{26} keto-ol (I)	HG_{26} keto-ol (II)	Combined HG_{26} keto-ols (I + II)	HG_{26} diol (I)	HG_{26} diol (II)	HDI_{26}
Elevation	r	0.042	-0.216	-0,853**	-0,382*	-0.043	-0.239	0.017	-0,400*	-0.041	0.109	0.131	0.364	0.198	0.238	-0.013	-0.152	0,362*	0,414**	-0.289	0.109	-0,492**
	p	0.797	0.175	0.000	0.014	0.788	0.132	0.918	0.011	0.798	0.522	0.441	0.080	0.416	0.145	0.951	0.344	0.020	0.007	0.067	0.498	0.001
	n	39	39	39	39	39	39	37	38	39	35	35	22	17	37	21	39	39	39	39	39	39
Lake Depth	r		0,349*	-0.106	-0.161	0.179	-0.031	0.110	-0.078	0.228	-0.228	-0,421**	-0.316	-0.384	-0.58	-0.232	-0.006	-0.079	0.046	0.135	-0.081	-0.059
	p		0.026	0.511	0.316	0.262	0.845	0.504	0.634	0.151	0.175	0.010	0.132	0.105	0.727	0.286	0.970	0.625	0.775	0.402	0.617	0.715
	n		39	39	39	39	39	37	38	39	35	35	22	17	37	21	39	39	39	39	39	39
Lake Surface Area	r			0.112	0.178	0.098	0,311*	0.150	0.209	0.169	-0.154	-0.191	0.219	-0.258	-0.154	-0.221	-0.275	-0.002	-0.115	0.206	-0.125	0.104
	p			0.487	0.266	0.540	0.048	0.361	0.195	0.290	0.364	0.259	0.304	0.285	0.348	0.310	0.081	0.990	0.475	0.196	0.437	0.519
	n			39	39	39	39	37	38	39	35	35	22	17	37	21	39	39	39	39	39	39
MAAT	r				0,488**	0.254	0,352*	-0.082	0,572**	0.021	-0.063	-0.064	-0,435*	-0.009	-0,402*	-0.189	-0.114	-0.073	-0.149	0,369*	-0.307	0,335*
	p				0.001	0.109	0.024	0.619	0.000	0.895	0.709	0.708	0.034	0.970	0.011	0.387	0.478	0.650	0.352	0.018	0.051	0.032
	n				39	39	39	37	38	39	35	35	22	17	37	21	39	39	39	39	39	39
BWT	r					0.266	0,539**	-0.196	0,545**	-0.106	0.149	0.191	0.116	0.328	-0,573**	-0.371	-0.201	0.129	-0.017	0.107	-0.166	0.086
	p					0.093	0.000	0.231	0.000	0.509	0.378	0.256	0.588	0.170	0.000	0.082	0.209	0.423	0.917	0.504	0.299	0.591
	n					39	39	37	38	39	35	35	22	17	37	21	39	39	39	39	39	39
SW pH	r						0,567**	0.088	0,596**	0.127	0.139	0.190	0.000	0.033	-0.244	-0.239	-0.213	0.183	0.231	0.198	-0.273	-0.123
	p						0.000	0.596	0.000	0.431	0.414	0.261	0.999	0.893	0.134	0.271	0.181	0.253	0.147	0.216	0.085	0.443
	n						39	37	38	39	35	35	22	17	37	21	39	39	39	39	39	39
BW pH	r							-0.075	0,683**	0.115	0.242	0.154	0.279	0.258	-0,431**	-0,493*	-0.308	0.097	-0.036	0.175	-0.167	0.067
	p							0.652	0.000	0.472	0.149	0.362	0.186	0.287	0.006	0.017	0.050	0.547	0.823	0.275	0.298	0.678
	n							37	38	39	35	35	22	17	37	21	39	39	39	39	39	39
SW DO	r								0.158	0.187	-0.209	-0.197	-0.224	-0,826**	0.090	-0.071	-0.072	0.093	0.140	0.148	-0.204	-0.153
	p								0.337	0.255	0.229	0.256	0.305	0.000	0.598	0.754	0.662	0.571	0.396	0.390	0.214	0.352
	n								37	37	33	33	21	16	35	20	37	37	37	37	37	37
BW DO	r									-0.002	-0.083	-0.043	-0.081	-0.037	-0,440**	-0.388	-0,372*	0.222	0.099	0.291	-0,360*	0.010
	p									0.992	0.628	0.805	0.714	0.884	0.006	0.067	0.018	0.169	0.545	0.068	0.022	0.951
	n									38	34	34	21	16	36	21	38	38	38	38	38	38
Conductivity	r										0.238	-0,456**	-0.316	-0,552*	0.004	-0.217	0.138	-0.022	0.115	0.083	-0.113	-0.112
	p										0.157	0.005	0.133	0.014	0.981	0.320	0.391	0.891	0.472	0.605	0.480	0.486
	n										35	35	22	17	37	21	39	39	39	39	39	39
TP	r											0,435**	0.179	0,951**	-0.230	-0.204	0.218	0.064	0.144	-0.279	0.152	-0.178
	p											0.007	0.403	0.000	0.183	0.350	0.195	0.708	0.394	0.095	0.368	0.292
	n											35	22	16	33	21	35	35	35	35	35	35
TN	r												0,738**	0,906**	-0.030	0.131	-0.002	-0.006	-0.071	-0.118	0.130	0.056
	p												0.000	0.000	0.865	0.552	0.993	0.972	0.675	0.488	0.442	0.074
	n												22	16	33	21	35	35	35	35	35	35
DOC	r													0.515	-0.149	-0.138	-0.032	-0.026	-0.205	-0.236	0.244	0.089
	p													0.059	0.497	0.623	0.882	0.903	0.337	0.268	0.250	0.679
	n													12	21	13	22	22	22	22	22	22
Chl a	r														-0.372	-0.634	0.111	0.166	0.059	-0.311	0.082	-0.094
	p														0.117	0.306	0.651	0.497	0.811	0.195	0.740	0.702
	n														17	9	17	17	17	17	17	17
TOC	r															0,857**	0.210	-0.301	-0.154	0.120	0.071	0.136
	p															0.000	0.199	0.062	0.350	0.466	0.666	0.410
	n															21	37	37	37	37	37	37
TON	r																0.393	-0,431*	-0.354	-0.055	0.283	0.360
	p																0.064	0.040	0.097	0.802	0.190	0.092
	n																21	21	21	21	21	21
HG_{26} keto-ol (I)	r																	-0,431**	-0.248	-0,367*	0.301	0.096
	p																	0.005	0.119	0.018	0.056	0.550
	n																	39	39	38	39	39
HG_{26} keto-ol (II)	r																		0,923**	-0.126	-0,485**	-0,850**
	p																		0.000	0.432	0.001	0.000
	n																		39	39	39	39
Combined HG_{26} keto-ols (I + II)	r																			-0.123	-0,494**	-0,900**
	p																			0.442	0.001	0.000
	n																			39	39	39
HG_{26} diol (I)	r																				-0,737**	0,464**
	p																				0.000	0.002
	n																				39	39
HG_{26} diol (II)	r																					0.171
	p																					0.284
	n																					39

* Correlation is significant at 0.05 level

** Correlation is significant at 0.01 level
